# Cardiotoxicity as a Possible Side Effect of Statins

**DOI:** 10.31083/j.rcm2401022

**Published:** 2023-01-11

**Authors:** Aleksey Chaulin

**Affiliations:** ^1^Department of Histology and Embryology, Samara State Medical University, 443099 Samara, Samara Region, Russia; ^2^Department of Cardiology and Cardiovascular Surgery, Samara State Medical University, 443099 Samara, Samara Region, Russia; ^3^Research Institute of Cardiology, Samara State Medical University, 443099 Samara, Samara Region, Russia

**Keywords:** side effect, cardiotoxicity, statins, cardiac troponins, highly sensitive cardiac troponins, mechanisms

## Abstract

According to current views, statins have a wide range of beneficial effects (lipid and non-lipid) on the cardiovascular system, so they are one of the most commonly used drugs for the prevention and management of patients with cardiovascular diseases. However, it is important to note that information about many beneficial effects of statins is contradictory. In addition, a number of side effects of statins, in particular, myotoxicity, hepatotoxicity, diabetogenic property, etc., may limit the possibility of using statins or even force doctors to cancel these drugs. Also, some concerns are caused by recent studies reporting cardiotoxicity of statins and increased serum concentrations of biomarkers of myocardial damage (highly sensitive cardiac troponins (hs-cTns)) in patients taking statins. This article discusses in detail the possible mechanisms of cardiotoxicity of statins and outlines the directions for further research in this area.

## 1. Introduction

Numerous clinical studies [1, 2, 3, 4, 5] and meta-analyses [6, 7, 8] indicate the high efficacy of statins for the prevention and treatment of patients with atherosclerosis and cardiovascular pathologies. The main mechanism of action of statins is based on the inhibition of a key rate-limiting enzyme (3-hydroxy-3-methylglutaryl-coenzyme A–reductase) involved in the biosynthesis of cholesterol and the subsequent formation of atherogenic low-density lipoproteins (LDL). A decrease in cholesterol and LDL levels is accompanied by a marked decrease in the risk of atherosclerosis and cardiovascular diseases and their complications (myocardial infarction, ischemic strokes, occlusive diseases of peripheral arteries, death from cardiovascular disease) [1, 4, 5, 6, 7].

In terms of effectiveness of lipid-lowering effect and safety, statins are second only to inhibitors of proprotein convertase subtilisin/kexin type 9 (PCSK9) [9, 10, 11]. This enzyme, which belongs to the serine class, is an important participant in the metabolism of LDL by regulating the density of LDL receptors in the hepatocytes cell membrane [12, 13]. However, despite the fact that some drugs that inhibit PCSK9 (monoclonal PCSK9 antibodies—alirocumab and evolocumab) have been approved by leading foreign authorities (U.S. Food and Drug Administration and European Medicines Agency) for practical use, in the near future, due to economic reasons, they are unlikely to have the same high prevalence as statins. Thus, the paper reports that the annual cost of treatment is 14,000–15,000 dollars, which is unprofitable. According to researchers, to achieve economic feasibility, the cost of drugs that inhibit PCSK9 should be reduced by at least 70% [14]. Since such a reduction in the cost of treatment is not expected in the near future, statins will continue to be the main lipid-lowering drugs for the majority of the population.

**Fig. 1.**
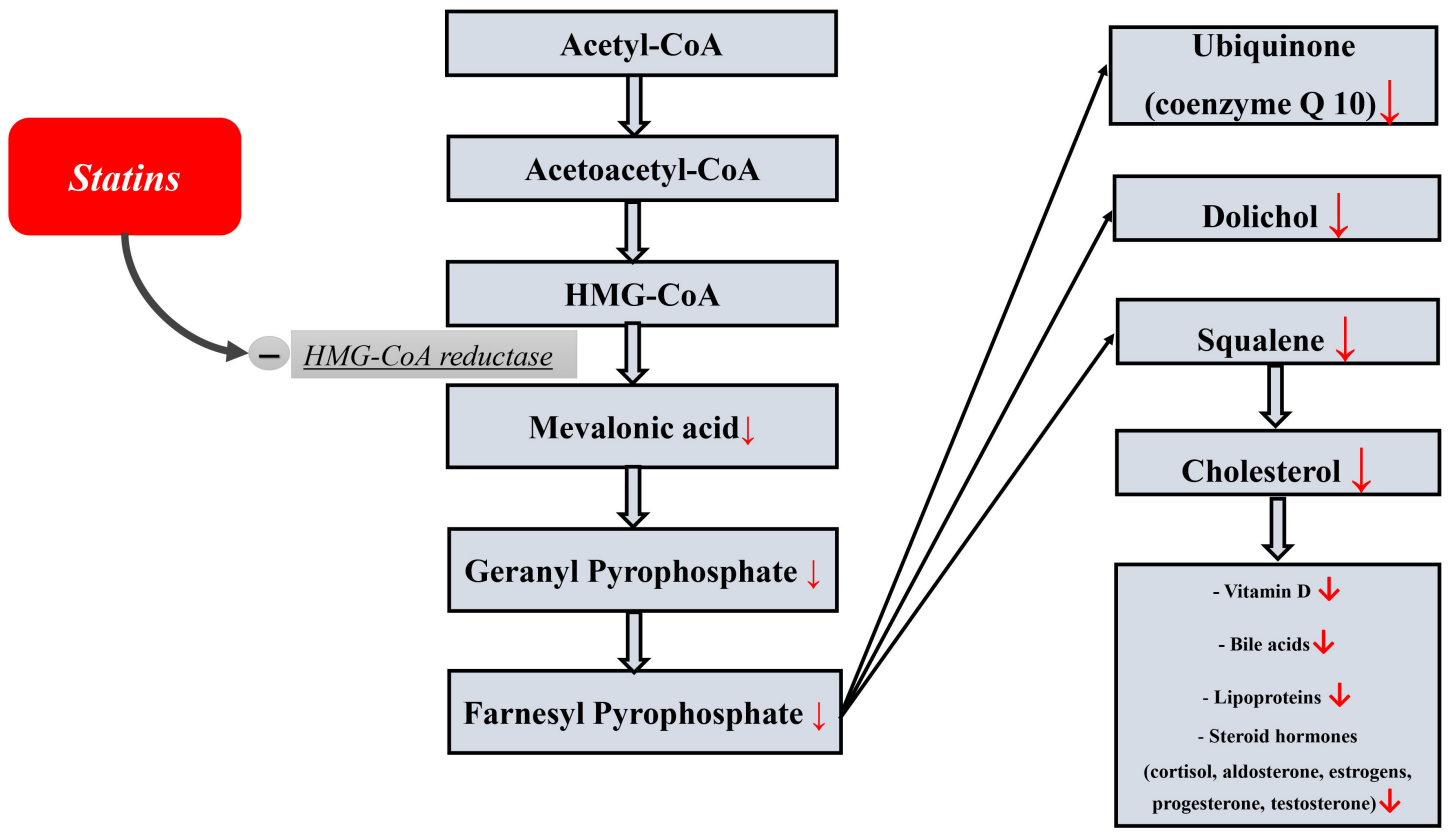
**The scheme of cholesterol biosynthesis and a number of important essential components from cholesterol**.

Statins, in addition to their main lipid-lowering effect (lowering cholesterol and LDL levels), have many additional (non-lipid) both favorable (pleiotropic) and toxic effects. The latter include hepatotoxicity, diabetogenic effect, myotoxicity, and a number of others [15, 16, 17, 18, 19]. The side effects of statins are largely due to the fact that cholesterol biosynthesis is extremely complex and involves many reactions and components of the metabolic pathway (Fig. 1) that can be important for the functioning of the body. The complete sequence of all cholesterol biosynthesis reactions was reproduced for the first time by the American biochemist R. Woodward, for which he was subsequently awarded the Nobel Prize in Chemistry [20]. Statins, by blocking cholesterol biosynthesis at the level of the enzyme 3-hydroxy-3-methylglutaryl-coenzyme A–reductase, can cause a deficiency of these substances necessary for the body. The latter include dolichol, vitamin D, bile acids, steroid hormones, lipoproteins, and ubiquinone (coenzyme Q), which is exceptionally important for all body cells, including myocardial cells. The following beneficial effects of coenzyme Q are most important for myocardial cells: it is an important component of the mitochondrial respiratory chain and is necessary for the production of energy in the form of adenosine triphosphate (ATP), antioxidant, and stabilization of cell membranes.

It is important to note that the mechanisms underlying pleiotropic and side effects are not studied sufficiently, and in many aspects there are a number of discrepant data. This is largely due to the design of ongoing studies and the different research methods used. As for the methods of study, they are constantly being improved, while opening up new possibilities for researchers and changing the understanding of many pathological and physiological processes occurring in the body. The most illustrative examples and confirmation of this in the context of this paper are laboratory diagnostic methods, in particular, immunochemical assays for determining cardiac troponins (cTns) in blood serum, as well as methods of molecular genetic and ultramicroscopic studies.

## 2. Cardiac Troponins as Early Biomarkers of Myocardial Cell Damage

The very first methods for determining cardiac troponin T (cTnT) and cardiac troponin I (cTnI) [21, 22] had extremely low sensitivity (high minimum detectable concentration 500–1000 ng/L and above) and could only detect large-focal myocardial infarctions late from the time of admission (12–24 hours or more), therefore, they were significantly inferior to the cardioenzyme total creatine kinase (CK) and CK-MB isoform, which was then used as the gold standard. However, as the sensitivity of troponin immunoassays increased, the minimum detectable concentration decreased significantly to only a few ng/L or less in modern high-sensitive (HS) immunoassays [23, 24, 25]. Thus, a newly developed assay provides the minimum detectable concentration of only 0.12 ng/L, which is more than a thousand times higher than the sensitivity of the very first troponin immunoassays and ten times more than HS 1st generation assays [26]. This allowed detecting serum cTns in all completely healthy patients. As a result, cTns are no longer considered strictly intracellular molecules, and provided that their concentration is less than the 99th percentile, they can be considered as normal metabolites of myocardial cells [23].

Moreover, HS methods for determining cTns have raised the issue of the need to take into account the gender, age and circadian characteristics of cTns concentration when using rapid algorithms for the diagnosis of acute coronary syndrome [23, 24, 27, 28, 29, 30]. Increased sensitivity has also contributed to the introduction of new data on the diagnostic capabilities of non-canonical to cardiovascular diseases biological fluids such as urine [31, 32], oral fluid [33, 34, 35, 36] and sweat [36] creating the foundation for further research on non-invasive diagnostic methods.

High-sensitive cardiac troponins (hs-cTns), apparently, can increase in blood serum under the effect of a number of drugs and biologically active substances that stimulate the myocardium, such as cortisol, catecholamines and adrenomimetics, thyroid hormones, which is explained by the fact that circadian fluctuations in cTns levels in healthy people coincide with circadian rhythms of endocrine glands producing these hormones [37, 38, 39, 40, 41, 42, 43]. Thus, myocardial cells began to be considered as cells, which are extremely sensitive to the effect of various factors.

## 3. Mechanisms of Statin-Induced Increase in cTns Level: Analysis of Research Results

Some drugs, such as statins, are believed to have beneficial effects on the myocardium of patients, and therefore their effects on cTns levels have not been widely studied. Therefore, the data of S. Unlü *et al*. [43] reported a significant effect of statins on hs-cTns levels. The study included 56 patients, of which 26 were taking statins (experimental group) and 30 people were not taking statins (control group). All study participants performed moderate physical exercise according to a established protocol. Blood samples were taken before exercise and 4 hours after. In those subjects who took statins and exercised, the concentration of hs-cTns increased significantly after 4 hours compared with initial indices (11.4 ± 15.2 ng/L versus 7.7 ± 12.6 ng/L, *p* = 0.004). In the control group of patients who did not take statins and did only exercise, the level of hs-cTnT was not increased after 4 hours (7.74 ± 5.7 ng/L versus 6.4 ± 3.5 ng/L, *p* = 0.664) [43]. Thus, the concentration of hs-cTns in the experimental group was significantly higher than in the control group, which indicates a direct dependence on the use of statins by patients.

In addition, in some patients taking statins, the kinetics of hs-cTns elevation met the criteria for acute coronary syndrome diagnostic without ST segment elevation, approved by the European Society of Cardiology (ESC) [43, 44, 45].

The results obtained by Ünlü *et al*. [43] differ from the data of other researchers [46, 47]. So, Trentini *et al*. [46] studied the effect of statin drugs on different types of muscle fibers. Changes in the concentrations of slow and fast isoforms of skeletal troponins (ssTnI and fsTnI, respectively) and total CK, as well as a marker of heart muscle damage—cTnI, were assessed. In patients taking statins, there was a specific increase in the concentration of fsTnI, but not ssTnI, which indicates that fast-twitch muscle fibers are most sensitive to the damaging effect of statins. In addition, subjects using statins often had a subclinical increase in fsTnI, but not in total CK or CK-MB, which indicates a higher sensitivity and diagnostic significance of fsTnI at the early stages of the statin myopathy development. CTnI levels did not undergo a significant change in the group of people taking statins, compared with people who did not take them, which additionally confirms the selectivity of the statins damaging effect specifically on skeletal muscle fibers [46]. Eijsvogels *et al*. [47] studied the effect of statins on cTnI concentration in marathon runners 1 and 24 hours after the finish. The use of statins did not significantly affect the amount of cTnI release (*p* = 0.47) or the number of runners whose cTnI level exceeded the diagnostic point to exclude acute coronary syndrome (57% versus 51%, *p* = 0.65). In addition, there was no significant association between statin dosages and cTnI release (r = 0.09, *p* = 0.65). Based on the results, the researchers concluded that marathon-induced increases in cTnI are not altered when using statins [47].

By analyzing the possible reasons for obtaining inconsistent results, it may be noted that there are differences in the design of the conducted studies, as well as methods for determining cTns. For example, the Eijsvogels [47] study participants were not examined for coronary heart disease, in contrast to the paper of Ünlü *et al*. [43]. In addition, in two studies by Trentini [46] and Eijsvogels [47], cTnI concentration was determined using moderately sensitive methods, while Ünlü used a HS method to determine cTnT. The time of taking the biological material in patients after physical activity also differed. So, for example, too early (after 1 hour) and too late (after 24 hours) time of taking the biological material, which was observed in the study by Eijsvogels [47], may not give an accurate idea of the cTns level, since the moment of cTns release is too early, and within a day, normalization of elevated cTns values in healthy patients is possible.

In addition, the sensitivity of conventional (moderately sensitive) methods for determining cTns may not be enough to detect subclinical damage to the myocardium by statins [46, 47, 48, 49], while HS methods are able to register the fact of even the most insignificant damage [43, 44, 45]. Thus, statin therapy against the background of additional moderate physical activity not only has an adverse effect on the myocardium, but can also affect the accuracy of acute coronary syndrome diagnosis according to modern ESC diagnostic algorithms when using hs-cTnT. On the other hand, it can be assumed that in the study of Ünlü [43] cross-reacted anti-cTnT antibodies with skeletal troponin isoforms, which increased due to the myotoxic effect of statins. A similar cross-reaction was also recently found in a study where patients with various myopathies but no obvious symptoms of cardiovascular diseases, there was an increase in hs-cTnT and hs-cTnI in 68% and 4% of cases, respectively [50]. At the same time, it is very remarkable that non-specific (cross) reactions are most characteristic of HS methods for determining cTnT, which was just the same and was used in the study by S. Ünlü [43]. This not only may explain the mechanism of the statin-induced increase in cTnT in this case, but also creates some concerns when using these HS immunoassays to diagnose acute coronary syndrome.

Another very interesting case of increased cTns levels after taking statins was described by English researchers Collinson and Kiely [51]. An elderly patient taking atorvastatin did not show any symptoms of acute cardiovascular diseases. However, hs-cTnT levels were significantly elevated at 55 ng/L (99th percentile = 14 ng/L). Serial measurements of hs-cTnT also revealed elevated concentrations of hs-cTnT (120 ng/L and 25 ng/L), but the concentrations of hs-cTnI were within the normal range all this time (5 and 7 ng/L at the level of the 99th percentile = 30 ng/L). In the absence of any significant cardiac symptoms of an ischemic nature, the researchers concluded that there was a false-positive increase in the level of hs-cTnT. In the process of further investigation by researchers, such causes as heterophilic antibodies and development of a macrotroponin system were excluded as possible false positive factors [51]. Thus, the most likely mechanisms for increasing hs-cTnT are either a direct cardiotoxic effect of atorvastatin on myocardial cells, or a false-positive reaction of anti-cTns antibodies with skeletal troponin T molecules, which are released from skeletal muscle fibers due to their damage by atorvastatin. However, it is also worth noting that the latter mechanism is opposed by the fact that the authors of the study used modern certified troponin immunoassays, for which there were previously no similar cases of false-positive (cross) reaction of diagnostic antibodies with skeletal troponins.

Damage to skeletal muscles under certain conditions (chronic renal failure [52], hereditary skeletal myopathies [52, 53], and some types of glycogenosis (Pompe disease) [54] may be accompanied by re-expression of cTns molecules in skeletal muscle fibers and, accordingly, lead to increase in the levels of these cardiospecific markers in serum by releasing the latter into the bloodstream. Hypothetically, it can be assumed that statin-induced skeletal muscle injury will also lead to extracardiac re-expression of cTns molecules. At the same time, it should be noted that this is a very controversial mechanism [55, 56] and some researchers refute it [50, 57].

In general, based on the analysis of the literature, the possible mechanisms for increasing cTns level can be summarized in Table 1 (Ref. [43, 50, 51, 52, 53, 54, 55, 56, 57]).

**Table 1.** **Probable mechanisms of increasing the concentration of cTns in blood plasma in patients taking statins**.

Mechanisms for increasing	Comment	References
Cardiotoxic effects of statins	cTns increase due to the direct cardiotoxic effect of statins on myocardial cells	[43, 51]
False-positive increase in cTns	cTns are increased due to the non-specific interaction of anti-cTns antibodies with skeletal troponin molecules that are released due to statin-induced skeletal muscle damage (statin-induced myopathy)	[50, 55, 56, 57]
Re-expression of cTns molecules in skeletal muscles	cTns molecules are re-expressed in damaged skeletal muscle due to the effect of statins and react with anti-cTns antibodies when released into the bloodstream.	[52, 53, 54]

cTns, cardiac troponins.

## 4. Potential Mechanisms of Statins Toxic Effects on Myocardial Cells

Almost since their invention, statins have demonstrated a pronounced anti-atherogenic effect by significantly lowering cholesterol levels and reducing the risk of development and progression of atherosclerotic cardiovascular diseases, in this connection, even the very idea of the possible negative effects of these drugs on the myocardium seemed extremely improbable. However, improved methods of laboratory diagnostics, histology and molecular biology have brought a number of new data and views on this aspect. Thus, several experiments have reported the presence of potential cardiotoxic properties in statins [58, 59, 60, 61, 62, 63, 64, 65]. Godoy *et al*. [58] studied ultrastructural changes in the myocardium of experimental animals under the action of statins (atorvastatin and pravastatin), and possible molecular mechanisms underlying the potential cardiotoxicity of statins. With long-term (7 months) oral administration of atorvastatin to rats, significant ultrastructural disorders of mitochondria were noted: swelling, change in size, displacement, physical separation of mitochondria from each other, which in normal cardiomyocytes are all joined together using mitochondrial contacts, form a chondriome like a three-dimensional network [58]. It is noteworthy that similar ultrastructural changes in mitochondria are also characteristic of skeletal muscle tissues during statin treatment [66, 67], and in the heart muscle they are considered as the main symptom of metabolic changes that often precede the development of cardiac dysfunction [67, 68, 69]. Another statin drug, pravastatin, did not cause these negative changes in the myocardium of rats after long-term administration [58]. These data may indicate a different degree of cardiotoxicity of certain classes of statin drugs or certain statins. In accordance with the physico-chemical properties, the group of statins is divided into two classes: hydrophilic and lipophilic (Table 2) [70].

**Table 2.** **Properties of lipophilic and hydrophilic statins**.

Hydrophilic statins (rosuvastatin, pravastatin)	Lipophilic statins (lovastatin, simvastatin, atorvastatin, fluvastatin)
They are well soluble in water and do not dissolve in lipid-containing media	They are well soluble in lipid-containing media and do not dissolve in water
They are absorbed more slowly in the intestine, as they pass poorly through cell membranes; protein carriers are needed to penetrate the cell	They penetrate well through cell membranes (lipid bilayer), therefore they are absorbed faster and widely distributed in various tissues
They are metabolized by membrane-bound cytochrome P450 enzymes in the liver before excretion from the body	They are excreted unchanged from the body with urine, so it is necessary to adjust the dose in case of impaired renal function

According to a number of researchers [67, 68, 69, 70], lipophilic statins have more pronounced cardiotoxic properties. This may be due to higher uptake of lipophilic statins by myocardial cells [71], induction of myocardial cell apoptosis [72] and more pronounced inhibition of ubiquinone biosynthesis, which is accompanied by increased oxidative stress of myocardial cells [73]. However, cardiotoxic properties in a number of experimental studies were not found in individual lipophilic statins. Thus, according to the results of experimental studies, only two lipophilic statins (atorvastatin and simvastatin) have pronounced cardiotoxicity [58, 60, 64, 65, 72, 74].

When studying the effect of statins on gene expression in cardiomyocytes, it turned out that atorvastatin, but not pravastatin, repressed the genes responsible for maintaining the integrity and proper functioning of mitochondria. In addition, researchers have found that atorvastatin, but not pravastatin, inhibits intracellular cardiac Akt/mTOR signaling [58], which, as was previously shown, regulate the physiological function of the heart and the survival of myocardial cells [59]. This raises the question of how long-term inhibition of mTOR upon administration of atorvastatin [58] will affect the integrity and survival of myocardial cells.

Atorvastatin also adversely affects the endoplasmic reticulum, causing endoplasmic reticulum stress *in vitro* and subsequent apoptosis of myocardial cells [58]. A study by Ghavami also reported that simvastatin induces endoplasmic reticulum stress and apoptosis in human atrial fibroblasts [74].

Bonifacio [64] found pronounced cardiotoxicity of simvastatin. The objects of the study were rodent cardiomyocytes and the hearts of male mice. The administration of simvastatin caused damage to the mitochondria of myocardial cells, increased expression of the marker of apoptosis (atrogin 1), increased apoptotic death of myocardial cells. The use of simvastatin for 21 days led to a decrease in heart mass by 5% and atrophy of myocardial cells.

Recently, Zhang [65] studied the effect of four statins (atorvastatin, lovastatin, rosuvastatin, pravastatin) on the viability of human cardiomyocytes. The researcher found that only atorvastatin induces ferroptosis of human cardiomyocytes. Atorvastatin has been shown to inhibit the viability of human cardiomyocytes in a dose-dependent manner, accompanied by a significant increase in intracellular iron ions, reactive oxygen species and lipid peroxidation. It is very noteworthy that the greatest degree of these pathophysiological changes was observed in the mitochondria of cells and caused mitochondrial dysfunction of myocardial cells. In addition, the use of statins was accompanied by an increase in the levels of cardiomarkers (CK-MB).

Zhu *et al*. [61] found that statin-induced depletion of membrane cholesterol in myocardial cells leads to a disruption in the architecture of the transverse (T) tubules, which are key structures for conjugation of myocardial contraction processes. In addition to remodeling of myocardial cells T-tubules, by studying the myocardium morphology in an experimental model (isolated perfused myocardium according to Langendorff), the researchers found a violation of the integrity of intercalated discs (intercellular connections) between myocardial cells. These changes confirm previously found evidence that low serum cholesterol levels are associated with cardiac arrhythmias and poor prognosis in patients with chronic heart failure [61].

Statins can also affect the uptake of glucose by myocardial cells, while pravastatin and rosuvastatin, in contrast, have a less significant effect on this process [60]. Inhibition of insulin-induced glucose uptake by myocardial cells by atorvastatin can lead to a decrease in myocardial aerobic metabolism, since glucose is one of the main energy sources for the myocardium.

Finally, as a final potential long-term mechanism of statin cardiotoxicity with respect to the myocardium, it is possible to consider the fact that statins increase the risk of developing diabetes mellitus [75, 76], which in turn subsequently leads to micro- and macroangiopathies, the development of chronic heart disease and gradual reduced delivery of blood and energy resources to myocardial cells [77, 78].

**Fig. 2.**
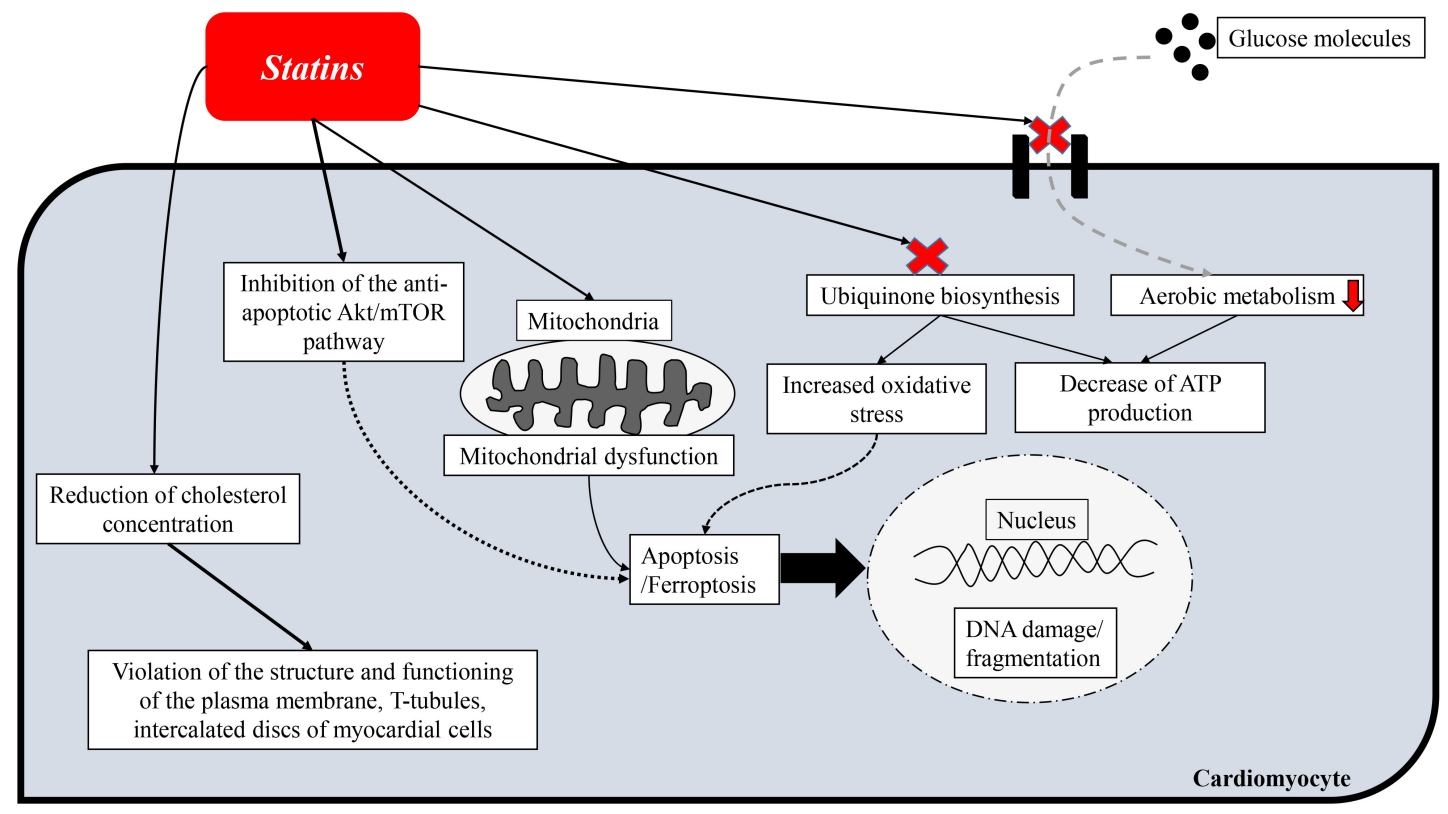
**Scheme of cardiotoxic effects of statins**.

Thus, statin-induced adverse myotoxic effects are characteristic of both skeletal and cardiac muscle tissue. Different statin drugs may have different effects at the molecular genetic and cellular levels, inducing or suppressing certain intracellular signals that may enhance beneficial effects on the myocardium, and, on the contrary, weaken them and cause adverse changes. More experimental and clinical studies are needed to better understand the pleiotropic effects of statins and their clinical significance, which will subsequently be important in making decisions about which statin to use under certain conditions. The main cardiotoxic effects of statins are summarized in Fig. 2.

## 5. Future Directions of Research

It is important to note that many researchers, on the contrary, found cardioprotective properties of statins, which was expressed by antioxidant [79], antiaptoptotic [80], anti-inflammatory [81, 82] and antifibrotic [83] and other effects. Due to these properties, many scientists recommend using statins as cardioprotectors for the management of patients suffering from cancer and taking cardioprotoxic chemotherapeutic agents [84, 85]. However, these beneficial effects of statins are contradictory and have not been found in many other experimental and clinical studies [86, 87, 88, 89, 90]. In particular, in a recent observational study by Palazhy *et al*. [86], statin treatment did not lead to a reduction in oxidative stress. In addition, in clinical practice, the side effects of statins often prevail over the beneficial ones, which creates the need to reduce the dose of a statin drug or cancel it [90].

Given the inconsistency and insufficient knowledge of the existing data on the cardiotoxicity of statins, I believe that further studies using experimental simulation methods are needed to confirm or exclude the cardiotoxic effects of statins: administration of various statin drugs with different dosages to laboratory animals, followed by a study of blood serum by HS methods for determining cTns [91, 92, 93], as well as assessing morphological changes in the myocardium at the ultrastructural level using electron microscopy. First of all, it is necessary to establish the exact mechanisms of increasing hs-cTns in patients taking statins and to confirm or refute the cardiotoxic effects of statins (oxidative stress, endoplasmic reticulum stress, mitochondrial dysfunction, induction of apoptosis and ferroptosis, deformation of insertion discs, inhibition of glucose transport into myocardial cells).

## 6. Conclusions

Based on the results of experimental studies, it can be assumed that statins, especially lipophilic statins (atorvastatin and simvastatin) have cardiotoxic effects. In general, the toxic effect of statins on myocardial cells is manifested by the following effects: oxidative stress, endoplasmic reticulum stress, mitochondrial dysfunction, induction of apoptosis and ferroptosis, deformation of insertion discs and inhibition of glucose transport into myocardial cells. In addition, significant concerns are caused by data on an increase in the serum concentration of hs-cTns (the most sensitive and specific criteria for myocardial cell damage) in patients taking statins. However, the exact mechanisms of increasing cTn levels have not been established and much information about the cardiotoxic/cardioprotective effects of statins is contradictory, so additional research is needed in this area.

## Abbreviations

ATP, adenosine triphosphate; LDL, low-density lipoproteins; PCSK-9, proprotein convertase subtilisin/kexin type 9; cTns, cardiac troponins; cTnT, cardiac troponin-T; cTnI, cardiac troponin-I; HS, high-sensitive; hs-cTns, high-sensitive cardiac troponins; hs-cTnT, high-sensitive cardiac troponin T; hs-cTnI, high-sensitive cardiac troponin I; ESC, European Society of Cardiology.
